# Impact of maternal antibodies and weaning stress on the replication and transmission of human H3N2 influenza A in piglets

**DOI:** 10.1128/jvi.01975-25

**Published:** 2026-03-27

**Authors:** Giovana Ciacci Zanella, Matias I. Cardenas, Carl R. Hutter, Celeste A. Snyder, Meghan Wymore Brand, Bailey Arruda, Daniel R. Perez, Tavis K. Anderson, Daniela S. Rajão, Amy L. Baker

**Affiliations:** 1 Virus and Prion Research Unit, National Animal Disease Center, USDA - ARS 57837 , Ames, Iowa, USA; 2 Department of Veterinary Microbiology and Preventive Medicine, College of Veterinary Medicine, Iowa State University 537366 , Ames, Iowa, USA; 3 Department of Population Health, College of Veterinary Medicine, University of Georgia 308501, Athens, Georgia, USA; 4 Interdepartmental Microbiology Graduate Program, College of Agriculture and Life Sciences, Iowa State University 117232 , Ames, Iowa, USA; University of Minnesota Twin Cities, Minneapolis, Minnesota, USA

**Keywords:** reverse zoonosis, vaccination, maternally derived antibodies, weaning, influenza A

## Abstract

Human seasonal H3N2 influenza A viruses (IAV) are repeatedly transmitted to swine. Modern indoor swine production facilitates close contact between human employees and pigs in their care during certain husbandry events, like weaning, that may increase the chance of interspecies transmission of IAV. This study investigated the effect of matched and mismatched maternal-derived antibodies (MDA) acquired from homologous and heterologous vaccinated sows on piglet susceptibility to a human-like H3N2 virus, generated to mimic the 2010.1-lineage H3N2 introduction from humans to swine. Additionally, we assessed the impact of weaning-related stress, along with immune status, on transmission to naïve direct-contact pigs. Seeder piglets from immune- and weaning-status groups were directly inoculated. Two days post-inoculation (DPI), naïve direct-contact pigs were placed with seeders. Nasal swabs were collected daily in seeder and contact pigs to evaluate shedding and transmission kinetics, and seeders were euthanized at 5 DPI to evaluate viral replication in the lower respiratory tract. Matched MDA was effective in reducing nasal shedding in challenged pigs and minimizing transmission to contacts, whereas mismatched MDA was not. Additionally, there was an increase in shedding and transmission in weaned pigs with mismatched MDA compared to littermates that remained on the sow. These results identify critical control points in swine production to implement practices for mitigating human-to-swine and swine-to-swine transmission to prevent the establishment of novel lineages in pig populations.

**IMPORTANCE:**

Defining the factors that increase the susceptibility of pigs to infection with human influenza A viruses (IAV) is critical to understand why those viruses transmit to the new host. IAV is frequently detected in nursing pigs, where it was shown that maternal-derived antibodies (MDA) may reduce clinical signs but may not prevent infection and transmission. Infected weaned piglets can then move viruses from the sow farm to offsite nurseries, where they can cause outbreaks with clinical disease as MDA wanes. Determining management practices that can be modified to reduce interspecies transmission of viruses to pigs is economically beneficial to the swine industry and could help define measures to prevent new spillover events. Reducing spillover of human IAV into pig populations also benefits public health by reducing genomic and phenotypic diversity in swine and the subsequent potential for zoonotic transmission.

## INTRODUCTION

Influenza A virus (IAV) is one of the most frequently detected swine respiratory pathogens and can cause outbreaks in swine herds throughout the year. Infected pigs develop acute respiratory disease, and the high morbidity in herds leads to significant economic losses for swine producers (1, 2). Although only H1N1, H3N2, and H1N2 subtypes are endemic in pigs, numerous genetically and antigenically diverse IAV lineages and clades circulate in swine worldwide (1, 3). This diversity arises from the ability of the virus to evolve through reassortment between different lineages and through mutations in the surface proteins hemagglutinin (HA) and neuraminidase (NA).

Transmission between swine and humans also contributes to the genetic and antigenic diversity of swine IAV (3–5). Frequent introductions of antigenically distinct human-origin strains into swine herds pose a significant barrier to infection control and the development and employment of broadly protective vaccines. Furthermore, mixing different levels and specificity of population immunity promotes the maintenance of endemic IAV strains in U.S. commercial swine herds (3). Influenza viruses notoriously cross species barriers, but to establish in a new host, there must be an initial inter- and intraspecies transmission period, followed by adaptation for efficient replication and transmission. Strains that are endemic in pigs can sometimes spill over to humans, leading to zoonotic infections. Swine-origin IAVs detected in humans, termed “variant viruses,” are reported often but rarely result in sustained human-to-human transmission (6, 7). In contrast, reverse-zoonotic transmission events from humans to pigs are frequent and lead to sustained transmission and establishment of new lineages (5, 8). All IAV viruses currently endemic in U.S. swine contain an HA gene that evolved from a human-origin virus (9–11). When human IAV spills over into swine and continues to circulate, the internal genes of the human-origin viruses are often replaced via reassortment with genes from endemic swine IAV strains, potentially contributing to adaptation. However, the human-origin HA and/or NA segments are maintained (8). This diversity creates a reservoir of viruses in swine that represent a zoonotic risk as they evolve and become antigenically distinct from the human seasonal strain that was introduced into swine.

One example of this phenomenon is the 2010.1 H3N2 swine IAV lineage. In 2012, genomic surveillance detected a novel reassorted IAV strain that contained human-seasonal H3 and N2 (12). These viruses reassorted with endemic swine IAV while maintaining the human-origin H3 segment, and this led to a new endemic lineage of swine IAV (2010.1 H3 lineage) (13). This lineage has since become one of the most frequently detected H3 subtype lineages in U.S. swine (3). The internal genes of the 2010.1 H3 HA either originated from a triple-reassortant H3N2 virus detected in swine populations in the 1990s (T) (14) or from the H1N1pdm09 (P) lineage (15). Despite recurrent human-to-swine IAV introductions (5, 8, 16–18), not all have persisted and established new endemic swine lineages (5, 8, 19, 20). The factors that facilitate the establishment of a human-origin virus in pigs are unclear, but given the frequency of human H3 detection in U.S. swine (21), understanding these factors will help determine whether a single detection represents a threat of permanently altering swine IAV diversity.

The immune response to endemic and novel viruses can affect the evolutionary trajectory of a pathogen (22–24). However, quantifying how variation in immune responses affects the frequency of interspecies transmission and subsequent viral adaptation to a new host species has not been systematically investigated. In U.S. commercial swine farms, herd vaccination is a common strategy to control IAV, with most vaccines administered to sows before farrowing so they can transfer maternally derived antibodies (MDA) to their piglets. These vaccines do not usually provide neutralizing protection against all IAV circulating in pigs, and the heterogeneous immune landscape can result in variable protection due to MDA mismatch to IAV that is circulating in different production locations (25, 26).

Stress is another critical factor to be considered in swine production that may influence virus transmission. Stress affects immune function and susceptibility to infectious agents in many species, including humans and pigs (27). In modern swine production, weaning is one of the most stressful events in a pig’s life and is associated with immune system dysfunction by alterations in immune cell recruitment and proliferation (28, 29). Weaning separates piglets from the sow, typically at 3 weeks of age, marking a significant change in their diet, environment, and immune development. In addition to the various stressors associated with weaning, this period is marked by increased human interaction, transportation, and commingling of a diverse population of piglets from other litters with differing influenza immunity and infection statuses (30). Stressed, weaned piglets with dysregulated immune responses may be more susceptible to viral infections (31), and consequently, stressed pigs may represent a more favorable host condition for sustained replication and transmission of non-adapted human-origin viruses.

In this study, we hypothesized that mismatched or lack of vaccination in the sows and the stress of weaning negatively impact the piglet’s immune responses, facilitating replication and transmission of a human-origin IAV. Moreover, we predicted that these standard management practices then facilitate the establishment of these viruses in the new host by allowing higher levels of viral replication and shedding compared to environments where optimal vaccination is in place and stress is minimized. To test this hypothesis, we generated a virus that mimicked the 2010.1 H3N2 human-to-swine spillover through reverse genetics for a pathogenesis and transmission study. This virus contained the HA and NA gene segments from a human seasonal H3N2 and the internal gene segments from endemic U.S. swine IAV representatives. This study determined how the host’s immune profile, specifically the transfer of maternal antibodies and stress, impacts the replication and transmission of IAV in novel hosts. Our work quantified the effect and interaction between matched, mismatched, and the absence of antibodies acquired from vaccinated sows and the weaning process on the susceptibility of piglets to a human-origin H3N2 IAV.

## RESULTS

### Antibody responses and serum cortisol levels

**Fig 1:**
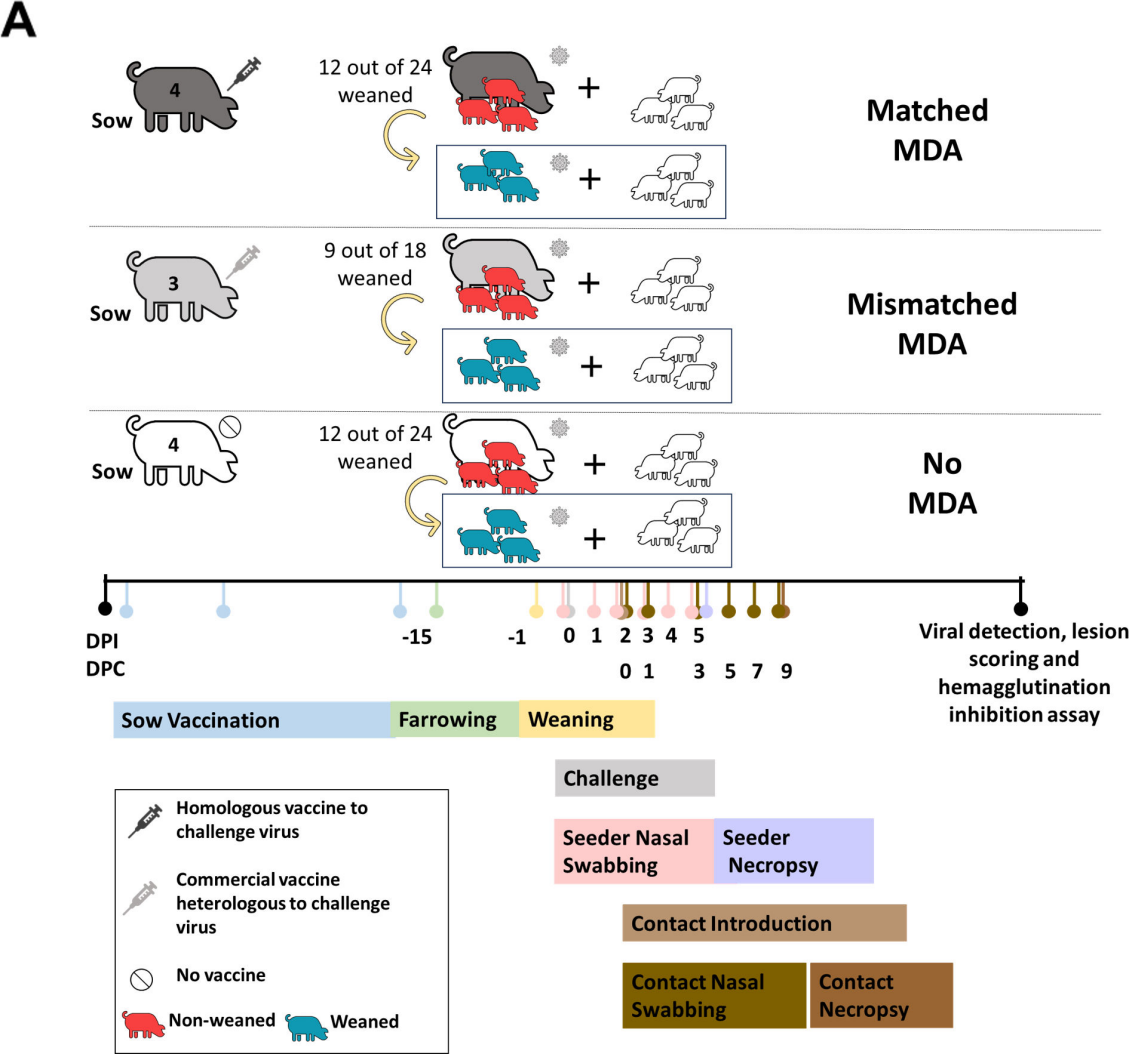
Animal study design. (**A**) Four sows were vaccinated with the hu-like H3N2rg whole-inactivated virus vaccine, three were vaccinated with the commercial Flusure XP, and four were not vaccinated for influenza A virus. Each vaccinated sow received three intramuscular doses; the first two were within 2 week intervals before artificial insemination, and the third and last dose was administered 1 month before farrowing. At 15 days post-birth, piglets with matched maternal antibodies, mismatched maternal antibodies, or no maternal antibodies were further subdivided into weaned or not weaned groups. Nasal swabs were collected daily. At 2 days post-inoculation (DPI), naïve direct-contact pigs were placed with seeders to evaluate transmission. Primary seeder piglets were humanely euthanized at 5 DPI and contact pigs at 9 days post-contact (DPC).

**Fig 2:**
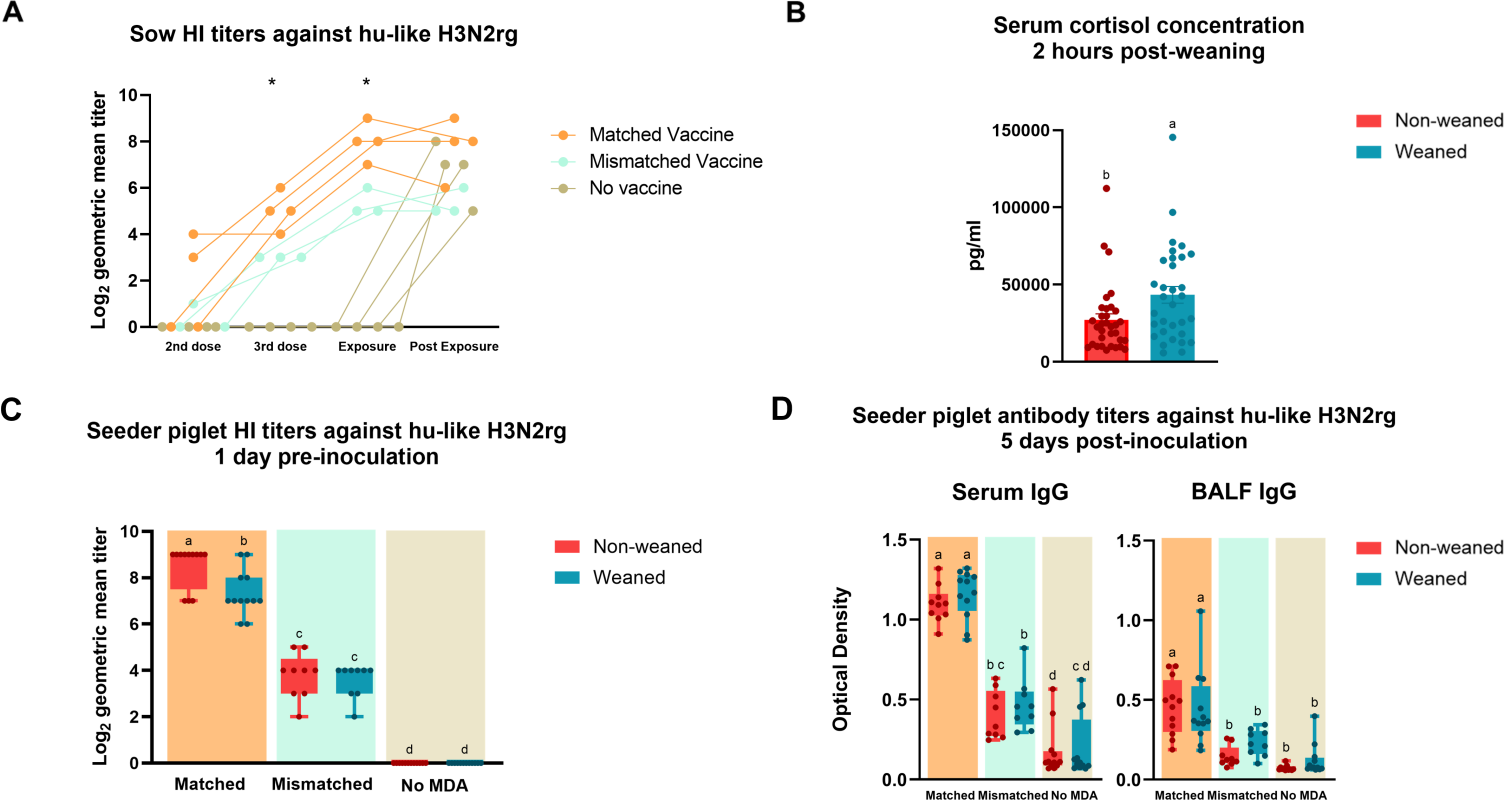
Antibody responses in sows and seeder piglets against hu-like H3N2rg. (**A**) Sow HI log_2_-transformed titers at the second and third vaccine doses, after exposure and post-exposure to hu-like H3N2rg virus. Sows received either a matched vaccine to the challenge virus (orange), a mismatched vaccine (green), or no vaccine (brown). Data represent the individual log_2_ reciprocal titers/10. (**B**) Serum cortisol concentrations from piglets 2 hours post-weaning assessed using a colorimetric competitive ELISA kit. Data are presented in picograms per milliliter. Piglets were either non-weaned (red) or weaned (blue). (**C**) Seeder piglet HI log_2_-transformed titers 1 day prior to inoculation. Data are stratified by maternal care group and presence or absence of MDA. Piglets were either non-weaned (red) or weaned (blue). (**D**) Seeder piglet serum and bronchoalveolar lavage fluid (BALF) IgG levels measured by ELISA 5 days post-inoculation. Data are presented as optical density (OD) values. Bars are showing all points. Asterisks (*) and different lower-case letters (a, b, c) indicate a statistically significant difference (*P* ≤ 0.05) by ordinary one-way ANOVA with Tukey’s multiple comparisons test (GraphPad Prism, GraphPad Software, La Jolla, CA).

We enrolled 11 bred sows in a vaccination program. Four sows received three doses of a homologous in-house whole-inactivated virus (WIV) vaccine containing the challenge virus (hu-like H3N2rg containing the A138S mutation in the HA, H3 numbering) while three sows received a commercial heterologous IAV vaccine. The remaining four sows were not vaccinated against IAV (Fig. 1). Sows farrowed, and piglets were monitored immediately after birth to ensure colostrum intake. Hemagglutination inhibition (HI) assays were performed against hu-like H3N2rg to assess vaccine-induced neutralizing anti-influenza antibodies and the transfer of maternal antibodies to piglets. Following the second vaccination, sows that received the homologous WIV vaccine developed significantly higher levels of antibodies than sows that received the commercial heterologous vaccine. This difference was measured in log_2_ reciprocal titers and persisted until the sows were exposed to piglets that had been inoculated with the virus (Fig. 2A). Naïve sows from the non-vaccinated group seroconverted after being exposed to their piglets that were challenged.

At 2 weeks of age, piglets were tagged and bled for evaluation of transfer of maternally derived antibodies. Piglets were divided into groups based on maternal-derived immunity and weaning status and entered the challenge and transmission phase of the study at approximately 3 weeks of age (Fig. 1). All piglets were bled to evaluate serum cortisol levels by a commercial ELISA assay 2 hours post-weaning and transport of three pigs per litter. Piglets that were weaned and transported to a different containment facility showed a significant (*P* = 0.03) increase in serum cortisol levels when compared to piglets that were maintained with their sow (Fig. 2B). Higher levels of serum cortisol in the weaned piglets compared to the piglets that remained with the sows demonstrated that the weaning process led to an elevated physiological acute stress response. However, statistical analysis did not find a significant correlation between nasal virus levels and serum cortisol levels, weaning status, or MDA immunity status (Data Set S1). HI assays performed on piglet serum samples at weaning demonstrated that they acquired MDA against hu-like H3N2rg through the ingestion of colostrum and milk (Fig. 2C). Piglets born to the matched WIV-vaccinated sows had robust HI titers against hu-like H3N2rg with an average of 7.9 log_2_. These piglets had significantly higher titers than those from sows vaccinated with the commercial mismatched vaccine (*P* = <0.0001) at weaning. As confirmed by NP ELISA (Data Set S1) and HI assays, piglets that ingested colostrum from non-vaccinated sows did not present anti-IAV antibodies at weaning.

One-day post-weaning, seeder piglets of different immune and weaning statuses were intranasally challenged with hu-like H3N2rg virus inoculum. Five days post-inoculation (DPI), serum and bronchoalveolar lavage fluid (BALF) samples were collected from seeder piglets during necropsy to evaluate systemic and mucosal anti-IAV IgG (Fig. 2D) and IgA (Fig. S1A) antibody levels. IgG antibodies against the hu-like H3N2rg challenge strain were evaluated by an in-house whole virus ELISA. This assay showed a similar pattern to HI pre- and post- challenge (Fig. S1B) with the matched piglets’ serum and BALF IgG antibody levels being statistically higher than both mismatched and no-MDA piglet groups. IgA levels were low in serum and BALF and not statistically different between the groups. These results suggest that most IAV antibodies transferred from these vaccinated sows to their piglets and escaped the gut were the IgG isotype. Weaning did not impact systemic or mucosal anti-IAV IgG and IgA antibody levels at five DPI.

### Viral shedding in seeder piglets

**Fig 3:**
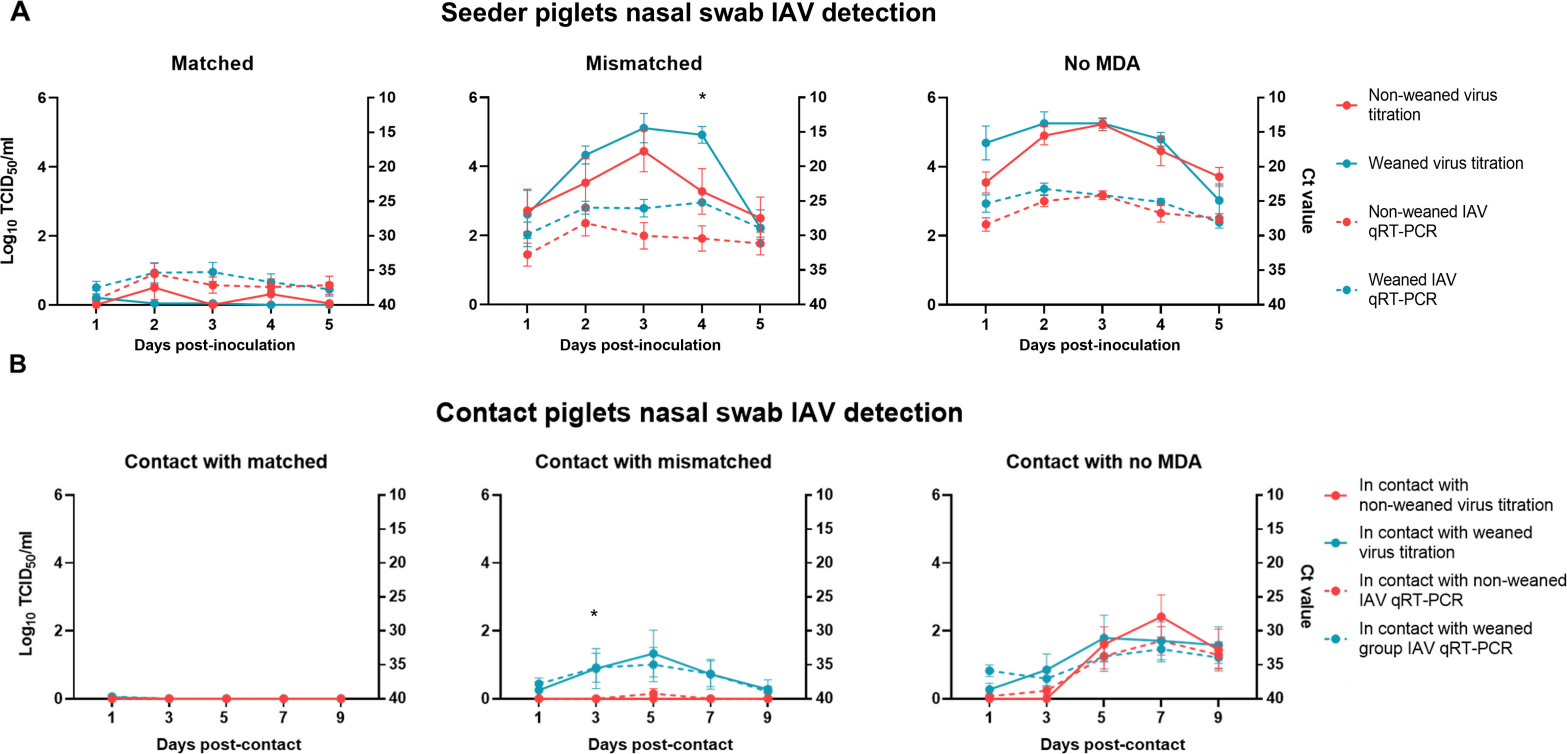
Viral detection in nasal swabs of seeder and contact piglets. (**A**) IAV titers and RNA detection from nasal swabs collected from seeder piglets daily for 5 days post-inoculation. Virus titration results (log_10_ TCID_50_/mL, solid lines; left *y*-axis) and qRT-PCR Ct values (dashed lines; right *y*-axis) are shown for piglets non-weaned (red) and weaned (blue) across matched, mismatched, and no-MDA groups. Asterisks (*) indicate titration data points that were significantly different between groups (*P* ≤ 0.05). (**B**) IAV detection in contact piglet nasal swabs collected at days 3, 5, 7, and 9 post-contact with seeder piglets. Virus titration (log_10_ TCID_50_/mL, solid lines; left *y*-axis) and qRT-PCR (dashed lines; right *y*-axis) results are displayed for contacts of seeder piglets in the matched, mismatched, and no-MDA groups. Each data point represents the mean ± standard deviation.

Nasal swabs were collected daily and BALF was collected at 5 DPI in the seeder piglets for the detection of IAV. No IAV was detected in the nasal swabs or BALF samples from the negative control piglets at any time point throughout the study (Data Set S1). IAV was detected by qRT-PCR in nasal swabs of 15 out of 24 matched MDA piglets, whereas all nasal swabs were positive in the mismatched MDA and the no-MDA piglets (Fig. 3A). Seeder piglets with matched MDA had significantly lower viral titers in nasal swabs than the mismatched MDA and no-MDA piglets on 1, 2, 3, 4, and 5 DPI (*P* < 0.0001) (Data Set S1), demonstrating that matched MDA acquired through the ingestion of colostrum from vaccinated sows protected the piglets against human-origin IAV, while mismatched MDA did not. Nasal swabs from the mismatched MDA seeder piglets had significantly lower viral titers than naïve piglets without MDA on days 1 (*P* = 0.004), 2 (*P* = 0.007), and 5 (*P* = 0.024) post-inoculation. There was a trend for weaned piglets in the mismatched MDA group to shed higher amounts of virus than the piglets that stayed with the sow (non-weaned), and this difference was statistically significant on day 4 DPI (*P* = 0.033 for log 10 transformed 50% tissue culture infectious dose [TCID_50_]/mL viral titer and 0.013 for Ct).

IAV was detected by qRT-PCR in only 1 BALF sample from piglets in the non-weaned matched MDA group but was negative for viral isolation, whereas 11 out of 18 piglets from the mismatched MDA and 18 out of 24 from the no-MDA groups were positive (Fig. S1B). There was a significant difference between the amount of virus isolated from the lungs of piglets with matched MDA (group average of 0) in comparison to those with no MDA (group average of 1.2). Matched MDA were protective against IAV replication in the lower respiratory tract in addition to the upper respiratory tract. Unexpectedly, most of the seeder piglets that were positive for IAV detection in their lung samples (66.7%) were non-weaned piglets in the mismatched or no-MDA groups. In addition, the non-weaned piglets from all immune statuses had significantly higher virus titers in their lungs (group average of 0.94 log) than those that were weaned (group average of 0.17) (*P* = 0.017). A significant relationship (*P* < 0.001) was found between local or systemic antibody levels (IgG and IgA) and the BALF Cts in only 35% of the samples. This indicates that antibody presence accounts for only ~one-third of the variation in Cts, highlighting the need to explore other factors that might be contributing to this phenomenon.

### Transmission to naïve direct-contact piglets

At 2 days post-inoculation of the seeder piglets, naïve contact piglets were placed in nose-to-nose contact with each group of seeder piglets to assess viral transmission across different immune statuses. Only 1 of 24 MDA group contacts had virus detected by qRT-PCR in the nasal swabs on 1 DPC in the weaned group but was negative for viral isolation (Fig. 3B). Six out of 18 mismatched MDA group contacts had IAV detected in their nasal swabs, and five of those were in contact with weaned piglets. Nineteen out of the 24 no-MDA group contacts had IAV detected in their nasal swabs, and 11 of those were in contact with weaned piglets. There was a trend for groups in contact with weaned piglets with mismatched MDA to have more pigs shedding, and at higher viral titers in nasal swabs than contacts with the piglets that remained with the sows (non-weaned), and this difference was statistically significant on day 3 post-contact (*P* = 0.040). The nasal swab viral titers of the contacts placed with matched MDA were significantly lower than those of the contacts placed with no-MDA piglets on days 5 (*P* < 0.001), 7 (*P* < 0.0001), and 9 (*P* < 0.001) post-contact (Data Set S1). Mismatched MDA contacts also had significantly lower nasal swab viral titers than no-MDA contacts on day 7 (*P* < 0.001) and 9 (*P* < 0.001) post-contact.

To assess seroconversion and confirm direct transmission from seeder piglets to the contacts, serum was collected from the contact piglets at 9 days post-contact and assessed by HI assay against challenge virus and NP ELISA (Table 1; Data Set S1). None of the contact piglets placed with the non-weaned matched piglet group had HI titers or NP antibodies detected at 9 DPC. For the piglets in contact with weaned matched MDA piglets, only 1 out of 12 contact piglets had a suspect HI titer of 1:20 against the hu-like H3N2rg challenge virus, and this same pig had a negative NP ELISA result. The piglets in contact with mismatched MDA piglets had one out of nine non-weaned and three out of nine that were weaned with suspect titer detected by HI, and only two in the weaned group had suspect values by NP ELISA. Five out of 12 piglets placed with each non-weaned and weaned no-MDA groups also showed signs of initial stages of seroconversion with low levels of neutralizing antibodies detected by HI. Only one piglet from the weaned group tested positive for NP antibodies and one with suspect results. There was no statistical difference in log_2_ geometric mean HI titers between the contact groups.

**TABLE 1:** Distribution of percentage positive, suspect, and negative seroconversion results to influenza A virus at 9 days post-contact among direct-contact groups

Group in contact with	Subgroup^*a*^	Hemagglutination inhibition^*b*^			NP ELISA^*b*^		
		Positive >40	Suspect 20	Negative ≤10	Positive <0.6	Suspect 0.6–0.7	Negative >0.7
Matched maternal-derived antibodies	Non-weaned (12)	0%	0%	100%	0%	0%	100%
	Weaned (12)	0%	8.3%	91.7%	0%	0%	100%
Mismatched maternal-derived antibodies	Non-weaned (9)	0%	11.1%	88.9%	0%	0%	100%
	Weaned (9)	22.2%	11.1%	66.7%	0%	22.2%	77.7%
No maternal-derived antibodies	Non-weaned (12)	0%	41.6%	58.3%	0%	0%	100%
	Weaned (12)	16.6%	25%	58.3%	8.3%	8.3%	83.3%

^*a*^ Numbers in parentheses are the total number of pigs in the group.

^*b*^ Percentage of pigs in each contact subgroup for each test category.

### Pathology

No macroscopic lesions typical of experimental IAV infection were observed in any of the lungs from seeder piglets at five DPI necropsy, consistent with a previous study (32). In the lung sections, minimal microscopic lung lesions were present, and seeder piglet scores were comparable to the negative controls. Average microscopic lesion scores of the lungs were minimal, and there was no significant difference between the scores of the MDA groups and weaning subgroups (Table 2). Microscopic tracheal lesions were more variable, and average scores ranged from minimal to severe. Piglets in the matched MDA group, independent of weaning status, had significantly fewer tracheal microscopic lesions than those from the no-MDA group (*P* = 0.0001), showing that matched MDA protected piglets against tracheitis. Mismatched MDA piglets had microscopic tracheal lesion scores that were not statistically different from other groups. When accounting for weaning, there was no significant difference between the piglets overall or within the immune status group. Immunohistochemical (IHC) NP antigen staining of the trachea sections complemented the microscopic lesion scores and the IAV detection in BALF samples by demonstrating that matched and mismatched MDA significantly reduced the number of antigen-positive cells in the lower respiratory tract of piglets after challenge when compared to naïve piglets. There was a trend for weaned piglets to have lower microscopic lesion and IHC scores in the trachea than non-weaned piglets within their immunity status groups; however, these trends were not statistically significant.

**TABLE 2:** Macroscopic and microscopic lesion scores in the lower respiratory tract of pigs inoculated with hu-like H3N2rg

Group	Subgroup	Percentage of macroscopic lung lesions^*a*^	Microscopic lesion scores		Immunohistochemical staining score
			Lung (0–20)	Trachea (0–8)	Trachea (0–4)
Negative controls		0.00 ± 0.00^a^	0.00 ± 0.00^a^	0.00 ± 0.00^a,b^	0.00 ± 0.00^b,c^
Matched maternal-derived antibodies	Non-weaned	0.00 ± 0.00^a^	1.33 ± 0.98^a^	1.08 ± 1.24^a^	0.42 ± 1.16^b,c^
	Weaned	0.00 ± 0.00^a^	1.00 ± 0.95^a^	1.08 ± 1.38^a^	0.00 ± 0.00^c^
Mismatched maternal-derived antibodies	Non-weaned	0.00 ± 0.00^a^	0.66 ± 0.50^a^	2.55 ± 1.94^a,b^	3.00 ± 1.73^a^
	Weaned	0.00 ± 0.00^a^	0.77 ± 0.44^a^	1.78 ± 1.99^a^	1.89 ± 1.90^a,b^
No maternal-derived antibodies	Non-weaned	0.00 ± 0.00^a^	1.50 ± 2.43^a^	4.64 ± 2.30^b^	3.6 ± 1.15^a^
	Weaned	0.00 ± 0.00^a^	1.33 ± 0.98^a^	2.75 ± 1.81^a,b^	2.5 ± 1.73^a^

^*a*^ Group weighted means ± standard error. Different lowercase letters within a column indicate statistically significant difference (*P* ≤ 0.05) by ordinary one-way ANOVA with Tukey’s multiple comparisons test) (GraphPad Prism, GraphPad Software, La Jolla, CA).

## DISCUSSION

While spillover events of human IAV to pigs occur, the factors that influence the virus’s ability to infect a new host and subsequently adapt and become endemic have not been quantified. The specific human-swine interfaces where transmission events occur most frequently are also not yet identified. We hypothesized that suboptimal sow vaccination and weaning stress negatively impact the piglet’s immune responses and facilitate the establishment of human IAV in the swine host by providing a more permissive environment for virus replication and transmission. To test this hypothesis and mimic the 2010.1 H3N2 human-to-swine spillover, a virus was generated by reverse genetics and used in a pathogenesis and transmission study (32, 33). This virus contained the HA and NA gene segments from a human seasonal H3N2 and the internal gene segments from endemic U.S. swine IAV representatives. Additionally, we introduced the A138S amino acid change in the HA that has been reported to allow swine-to-swine transmission (32). Seeder piglets were divided by MDA immune status (suckled on IAV-naïve sows or sows vaccinated with a matched or mismatched IAV vaccine) and weaning status (weaned or non-weaned) and intranasally inoculated at 3 weeks of age. Two days post-inoculation, naïve nose-to-nose contact pigs were placed in the rooms with seeders to evaluate transmission.

Piglets without MDA were the most susceptible to human-origin IAV. They shed high amounts of virus each of all 5 DPI and had the highest microscopic lesion and antigen-in-tissue detection scores. Piglets that nursed sows that received homologous vaccination presented high levels of neutralizing MDA against the challenge virus at all blood collection time points. The matched MDA effectively reduced nasal shedding in the piglets after the challenge and significantly reduced the transmission of the human-like H3N2 to direct contacts. ELISAs demonstrated that these antibodies were primarily IgG and were detected at higher levels systemically than in BALF after challenge. Antibody levels correlated with protection against infection, replication, and lesions in the upper and lower respiratory tract. The piglets that received colostrum from sows vaccinated with the commercial heterologous vaccine had intermediate neutralizing antibody levels that were insufficient to protect from infection against the human-origin IAV. These mismatched MDA piglets shed higher amounts of virus in their nasal secretions comparable to levels in the naïve piglets. When piglets’ IAV-specific MDA lack cross-reactivity to a strain that subsequently infects them, there may be a risk of vaccine-associated enhanced respiratory disease (VAERD) (34). Though the present study included an antibody mismatch group, there was no evidence of VAERD in the macroscopic lesion scores, likely due to cross-reactivity, albeit reduced in titer.

In the USA, 81% of large (>500 sows) breed-wean farms reported IAV sow vaccination either at pre-farrow or whole-herd vaccination (35, 36). Previous studies have shown that vaccination of gilts and sows can reduce endemic swine IAV transmission and clinical disease among the piglets. It is well established that strain-specific mass vaccination can decrease IAV shedding at the breeding herd level and the nursery (35, 37–40) and delay the time to become IAV-positive at the growing-finishing stage (41). However, these studies did not evaluate the impact of mismatched MDA or stress on the transmission of endemic swine IAV. Our results confirm that the presence of matched MDA in piglets prevented infection with a human-origin IAV, while MDA derived from suboptimally matched swine IAV vaccines did not. The heterologous cross-protection was further reduced by weaning stress, highlighting that current vaccination programs in the United States may not be efficient in preventing interspecies transmission of IAV or incursions of other novel IAV to a sow farm. Higher titers of IAV in the lungs of mismatched and no MDA pigs that stayed on the sow contrasted the nasal shedding results. The cause of this observation is unknown but may be due to the sows becoming infected and contributing to the viral load in the environment. It may also be due to the physiology of suckling milk from contaminated udders. Unfortunately, we did not collect nasal swabs or udder wipes to directly assess viral shedding and detection in the sows, but all sows in the non-vaccinated group seroconverted to the challenge virus after exposure, so it can be inferred that they were infected by virus shed from the piglets and may have served as an additional viral source. In this context, the non-vaccinated or mismatched vaccinated sows may have influenced infection dynamics in a way that was not fully captured within the experimental timeframe and with the samples collected. Regardless, this finding also supports the use of well-matched vaccines and application strategies that maintain robust cross-reactive antibody titers in sows.

IAV circulation in pig farms enhances the risk of antigenic drift and the emergence of new genotypes (42, 43). Piglets often act as reservoirs by maintaining and transmitting IAV within and between breed-wean farms when weaned and moved to other locations. Therefore, reducing IAV infection at weaning should decrease transmission between farms (35), lower prevalence of endemic swine IAV, and reduce risk of reassortment with newly introduced human-origin strains. We found that weaning stress increased piglet susceptibility to IAV and subsequent viral shedding and transmission in the mismatched MDA group. On day 4 post-inoculation, the weaned mismatched MDA piglets shed an average 42 times higher TCID_50_/mL viral titer compared with piglets in the same group that stayed with the sows. This upsurge in viral shedding could result in increased viral transmission among pigs if translated into field conditions.

In the U.S. pork industry, production practices such as weaning, transportation over long distances, and mixing piglets from different litters or even source herds are embedded in modern swine agricultural practices. Our results suggest that minimal modifications to some of these production practices during the pre- and post-weaning periods can mitigate human-to-swine IAV transmission and prevent the establishment of novel lineages in U.S. swine herds. Measures like matching sow vaccines to circulating strains to increase herd immunity before farrowing, maximizing colostrum intake by all piglets in the litter to create homogenous population immunity, and decreasing stressors related to weaning could decrease piglet susceptibility to human-origin IAV. Some of these production practices have also been shown to control endemic swine IAV effectively in field conditions, and our data confirm that there are production practices that can also minimize interspecies transmission probability. This process, however, is challenged by a need for farm- or region-specific genomic surveillance data to choose representative strains for custom vaccines, and once selected, the logistical and financial challenges associated with a comprehensive vaccination program across multiple farms (44). In addition, colostrum production by the sow and colostrum intake by the individual piglet are highly variable. Some of these identified risk factors may be difficult to standardize, and ongoing virus circulation and evolution in the field represent a continual problem (41, 45). Hence, maximizing sow immunity and MDA levels in piglets along with measures that reduce exposure to human-origin viruses during weaning and at the nursery should be taken as strategies for controlling human-origin IAV spillovers. Previous field research has shown evidence that workers often report to work infected with IAV of human origin that could potentially be transmitted to the pigs (46, 47). Improving worker policies and implementing consistent and effective biosecurity practices at the swine-human interface with effective policies are the most likely and cost-effective methods to prevent transmission. These policies could include restricting the entry of visitors, restricting access for those with influenza-like illness, enforcing farm worker sick leave, increasing IAV seasonal vaccination coverage, implementing shower-in/out policies, routine hand washing, and the use of personal protective equipment that includes face masks or respirators and gloves (40, 46, 48), and having more stringent control in farrowing rooms and for individuals that are processing piglets.

Weaning IAV-negative piglets presents an advantage to swine producers because of the benefits for pig long-term health, productivity, and well-being as well as improved immune responses to IAV vaccines (48). We provide evidence that sow vaccination and weaning stress contribute to piglet susceptibility to human-origin IAV in an experimental setting. Our results are limited to the experimental conditions, but stress is ubiquitous in agricultural swine production. In situations where piglets are stressed due to weaning, long-distance transportation, heat stress, and if any of these experiences are prolonged, the impact may be more dramatic than in our controlled experiment. An additional consideration is that we artificially limited the number of pigs within experiment rooms; in a field situation, stress may also be mediated by stocking density which tends to be much higher. Despite the limitations of our system, this study demonstrated that higher susceptibility and transmission rates due to mismatched sow vaccination and weaning stressors increased the likelihood of a human seasonal virus establishing in young pigs. Surveillance in swine must continue to be a priority for animal and public health, with priority given to specific animal–human interfaces that promote greater contact between pigs and people.

## MATERIALS AND METHODS

### Virus and vaccine preparation

A reassortant H3N2 human virus (herein referred to as hu-like H3N2rg) was generated using an eight-plasmid reverse genetics system as previously described (49). The virus contained the HA and NA gene segments from A/Victoria/361/2011 (H3N2) (VIC11), a human seasonal H3 isolate, five internal gene segments from a triple reassortant swine-origin H3N2 strain, A/turkey/Ohio/313053/2004 (H3N2) (OH04), and the matrix gene from the 2009 pandemic virus A/California/04/2009 (H1N1) (CA09). Additionally, the A138S mutation in the HA (H3 numbering) was introduced by site-directed mutagenesis, which has been demonstrated in previous studies to favor replication in swine lower respiratory tract cells and enhance transmissibility in pigs. The virus was propagated in Madin-Darby canine kidney (MDCK) cells to a maximum of three passages following rescue, with Opti-MEM (Life Technologies, Waltham, MA) containing antibiotics/antimycotics and 1 µg/mL of tosyl sulfonyl phenylalanyl chloromethyl ketone (TPCK)-trypsin (Worthington Biochemical Corp., Lakewood, NJ). Clarified virus from infected cell culture was diluted in phosphate-buffered saline (PBS) to a desired titer of 1 × 10^5^ TCID_50_/mL for use in the intranasal challenge.

To generate a WIV adjuvanted vaccine, the clarified virus was sucrose purified by ultracentrifugation at 20,000 RPM for 2 hours at 4°C (Optima XPN, Beckman Coulter, Brea, CA) and then inactivated by UV irradiation (UV Stratalinker1800, Stratagene; Agilent Technologies, Santa Clara, CA) for two rounds of 90 seconds duration each. Inactivation of the virus was confirmed by failure of the virus to replicate in MDCK cells. A commercial adjuvant (Emulsigen D; MVP Laboratories, Inc., Ralston, NE) was added at a 1:5 ratio. Each dose (2 mL) of the WIV contained approximately 256 HA units of the virus. The heterologous vaccine was the commercially available Flusure XP (Zoetis, Parsippany, NJ), a freeze-dried preparation rehydrated with Amphigen, that contains isolates from H1N1, H1N2, and H3N2 Clusters IV-A and B.

### Transmission study at weaning

The study design is shown in Fig. 1. Eleven sows (primiparous and multiparous) were obtained from a high-health herd considered free of IAV and porcine reproductive and respiratory syndrome virus. Sows were housed in isolation from other animals and cared for in compliance with the Institutional Animal Care and Use Committee (IACUC) of the National Animal Disease Center (NADC). Four were vaccinated with the hu-like H3N2rg WIV, three were vaccinated with the commercial Flusure XP, and four were not vaccinated for IAV. Each vaccinated sow received three intramuscular doses; the first two were within 2 week intervals before artificial insemination, and the third and last dose was administered 1 month before farrowing. All sows and gilts received a dose of Farrowsure Gold (Zoetis, Parsippany, NJ) before entering breeding protocol to prevent reproductive failure. Sows were moved to farrowing crates in animal biosafety level 2 (ABSL-2) 1 week before their due dates and delivered their piglets without surgical intervention.

Piglets were monitored carefully immediately after birth to ensure colostrum intake. Piglets were tagged and bled for evaluation of transfer of maternally derived antibodies at 2 weeks of age. All except three piglets per sow were weaned at 3 weeks of age and moved to a different animal building. Three piglets per sow were assigned to weaned seeder groups. Blood was collected 2 hours after weaning and transport to evaluate serum cortisol levels. Pigs were demonstrated to be free of influenza virus before challenge by nasal swab sampling (MagMax Viral RNA isolation kit and VetMAX Gold SIV Detection Kit, Thermo Fisher Scientific, Waltham, MA). Those born to non-vaccinated sows and gilts were shown by an IAV nucleoprotein (NP) blocking ELISA (Swine Influenza Virus Antibody Test, IDEXX, Westbrook, ME) to be free of anti-influenza virus antibodies. All animals were housed in ABSL-2 containment during the challenge phase of the study and cared for in compliance with the IACUC of the NADC. One day post-weaning, 72 3-week-old seeder piglets of different MDA (suckled on IAV-naïve sows or sows vaccinated with a matched or mismatched IAV vaccine) and weaning statuses were intranasally challenged with a 1 × 10^5^ TCID_50_/mL dose of the hu-like H3N2rg virus inoculum. At 2 DPI, 66 3-week-old naïve contacts were placed in direct nose-to-nose contact with each group of challenged piglets.

Nasal swabs were collected on 1–5 DPI from seeder piglets and on 1,3, 5, 7, and 9 DPC for the naïve contacts. Intranasally challenged seeder piglets were bled and humanely euthanized at 5 DPI and contacts at 9 DPC with a lethal dose of pentobarbital (Fatal Plus; Vortech Pharmaceuticals, Dearborn, MI). Lungs were aseptically removed, evaluated for macroscopic lesions, and lavaged with 50 mL of MEM containing 1% bovine serum albumin (BSA) to obtain BALF. Sections of the right middle or affected lung lobe and distal trachea were collected and fixed in 10% buffered formalin for histopathologic examination and scoring.

### Serology and viral detection

Serum and BALF samples collected throughout the study were evaluated for anti-influenza antibodies either by the commercial NP ELISA kit (Swine Influenza Virus Antibody Test, IDEXX, Westbrook, ME) (serum only), an isotype-specific IgG and IgA ELISA (serum and BALF), or by HI assays (serum only). For the commercial NP ELISA assay, samples were tested according to the manufacturer’s instructions. For the isotype-specific IgG and IgA ELISA, samples were tested as previously described with modifications (50). Modifications were as follows: the serum heat inactivation time was reduced to 10 minutes and the secondary antibodies used were Goat anti-Pig IgA and IgG Heavy Chain Antibody HRP Conjugated (Bethyl Labs and Fortis Life Sciences, Boston, MA). The influenza antigen used to coat the isotype-specific IgG and IgA ELISA plates was the challenge virus hu-like H3N2rg. For the HI assays, sera were incubated with receptor destroying enzyme (RDE) overnight at 1 serum:3 RDE in saline ratio. After RDE treatment, sera was inactivated at 56°C for 30 minutes, then incubated with 20% kaolin in PBS solution for 20 minutes, adsorbed with 0.5% and subsequently 100% turkey red blood cells (RBCs) for 20 minutes each to further remove non-specific hemagglutinin inhibitors and natural serum agglutinins. Turkey RBCs and virus dilutions were treated with NA inhibitor oseltamivir to prevent NA-mediated agglutination. Sow sera were tested against hu-like H3N2rg antigen and a surrogate Flusure XP strain A/swine/New York/A01104005/2011 [NY/11] (Data Set S1). At the same time, piglet sera were tested against only the challenge virus hu-like H3N2rg. HI assays were performed using standard techniques, and titers transformed by log_2_ scale and geometric group means calculated (3).

Serum cortisol concentrations from piglets 2 hours post-weaning were assessed using a colorimetric competitive ELISA kit (Enzo Life Sciences, Inc., Farmingdale, NY, USA) according to the manufacturer’s instructions with the addition of the steroid displacement reagent in a 1:99 ratio and an initial serum dilution of 1:8. Plates were read by SpectraMax M5 Multi-Mode Microplate Reader (Molecular Devices, LLC., CA, USA). Cortisol levels were calculated using a standard curve established from cortisol kit standard samples.

All nasal swabs and BALF samples were tested for IAV RNA by qRT-PCR (MagMax Viral RNA Isolation Kit and VetMAX Gold SIV Detection Kit, Thermo Fisher Scientific, Waltham, MA), following previously validated standard protocols (51). Samples with cycle threshold (Ct) values below 37 were subjected to virus isolation in 48-well plates containing confluent MDCK cells, in infection media and cultured at 37°C for 72 hours. Plates were fixed when the cytopathic effect reached 80% of cells on the positive control well and stained for immunocytochemistry (ICC) staining as previously described (52). Virus isolation-positive nasal swabs and BALF samples were tittered in 96-well plates of confluent MDCK cells in 100 µL 10-fold serial triplicate dilutions. At 48 hours post-inoculation, plates were fixed and stained (52). Titers were calculated for each sample as TCID_50_ per milliliter and transformed to log_10_.

### Pathology

During necropsy, the percentage of affected surface area per lung lobe was recorded and used to calculate a weighted macroscopic lung lesion score (53). Tissues fixed in 10% buffered formalin were transferred to 70% ethanol after 48 hours. Routine histologic procedures were used to process lung and tracheal tissues, and the slides were stained with hematoxylin and eosin. Microscopic lesions were evaluated and scored by a veterinary pathologist blinded to the group with parameters previously described (54–56). IHC staining was carried out manually on 5 µm-thick tracheal sections, using a primary antibody that targets the IAV nucleoprotein (NP) as previously described (57). IHC scores (0–4) were based on the amount of antigen-positive cells per scored section with 0: none, 1: 1–5, 2: 6–10, 3: 11–20, and 4: >21 cells.

### Statistical analysis

Macroscopic and microscopic pneumonia scores, HI titer log_2_ geometric means, log_10_-transformed nasal swab, and BALF viral titers were analyzed using analysis of variance (*t*-test (for two groups) and ordinary one-way ANOVA (for three or more groups) with Tukey’s *post hoc* multiple comparisons test of parameters with statistical differences. Comparisons were made between the challenged groups at the same time point using a 5% significance level (*P*-value <0.05) to indicate statistically significant differences. Linear regression was performed between antibody quantities and CT using the lm() function in R (R Development Core Team 2025). The test was performed on the whole data set and then on the swine weaned groups separately. The association between serum cortisol levels, litter origin, maternal immunity status, weaning status, and virus shedding and transmission outcomes was analyzed by multiple linear regressions in a multiple variable analysis. For this, average nasal swab Ct values from all five DPI were selected as the outcome variable: GraphPad Prism, GraphPad Software, La Jolla, CA).

## Data Availability

Clinical data associated with this study are available for download from the USDA Ag Data Commons under https://doi.org/10.15482/USDA.ADC/30043813 and https://catalog-beta.data.gov/dataset/impact-of-maternal-antibodies-and-weaning-stress-on-the-replication-and-transmission-of-hu.

## SUPPLEMENTAL MATERIAL

Data Set S1 jvi.01975-25-s0001.xlsx Data and statistics for Fig. 2, 3, and S1.

Figure S1 jvi.01975-25-s0002.tif Seeder piglet HI log2 transformed titers, IgA levels in serum and BALF samples, and BALF virus titration and qRT-PCR results.

Supplemental legend jvi.01975-25-s0003.pdf Descriptive legend for Fig. S1.

ASM does not own the copyrights to Supplemental Material that may be linked to, or accessed through, an article. The authors have granted ASM a non-exclusive, world-wide license to publish the Supplemental Material files. Please contact the corresponding author directly for reuse.
