# Supplementary figures and images for: Impact of maternal antibodies and weaning stress on the replication and transmission of human H3N2 influenza A in piglets
Source: J Virol. 2026 Mar 27;100(4):e01975-25. doi: 10.1128/jvi.01975-25 (PMC13098221; doi:10.1128/jvi.01975-25)

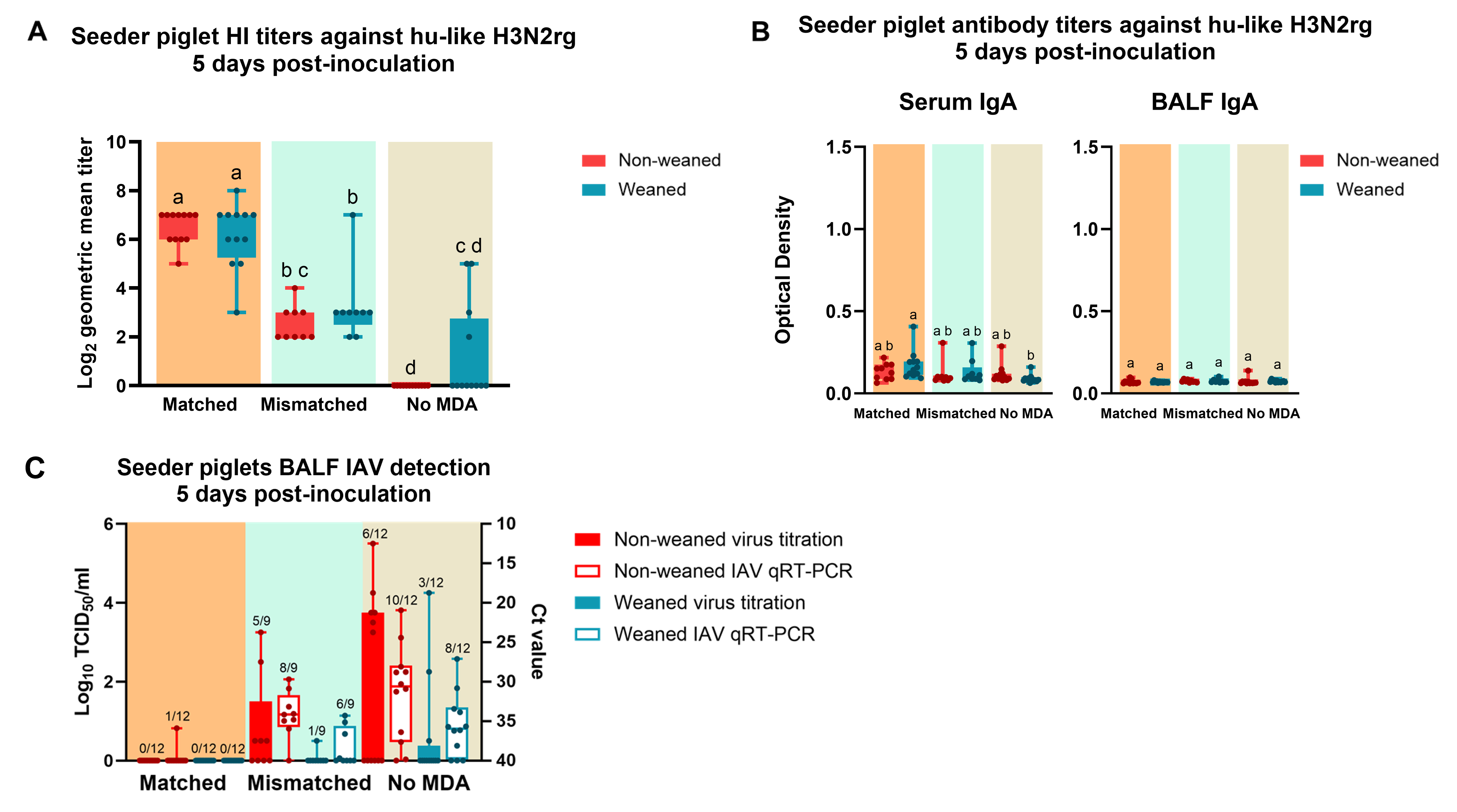

Supplement: Figure S1 — Seeder piglet HI log2 transformed titers, IgA levels in serum and BALF samples, and BALF virus titration and qRT-PCR results. [file jvi.01975-25-s0002.tif]
