# Supplementary material for: Impact of maternal antibodies and weaning stress on the replication and transmission of human H3N2 influenza A in piglets
Source: J Virol. 2026 Mar 27;100(4):e01975-25. doi: 10.1128/jvi.01975-25 (PMC13098221; doi:10.1128/jvi.01975-25)
Supplement: Supplemental legend — Descriptive legend for Fig. S1. [file jvi.01975-25-s0003.pdf]

Supplemental Figure 1. (A) Seeder piglet HI log2 transformed titers five days post-inoculation. Data is stratified by maternal care group and presence or absence of maternally derived antibodies (MDA). Piglets were either non-weaned (red) or weaned (blue). (B) Seeder piglet IgA levels in serum and BALF samples collected at five days post-inoculation, determined by ELISA. Data are shown as OD values for each group. Non-weaned group (red) and weaned group (blue). Different lower-case letters (a,b,c) indicate statistically significant difference ($p \leq 0.05$) by ordinary one-way ANOVA with Tukey's multiple comparisons test (GraphPad Prism, GraphPad Software, La Jolla, CA). (C) Seeder piglet BALF virus titration (log10 TCID50/ml; solid filled box; left y-axis) and qRT-PCR results (open box; right y-axis) at 5 DPI. Non-weaned group (red) and weaned group (blue). Numbers above the error bars show the number of positive pigs/total pig numbers in the group.
